# Cardiovascular mortality by cancer risk stratification in patients with localized prostate cancer: a SEER-based study

**DOI:** 10.3389/fcvm.2023.1130691

**Published:** 2023-08-04

**Authors:** Zehao Luo, Kaiyi Chi, Hongjun Zhao, Linglong Liu, Wenting Yang, Zhijuan Luo, Yinglan Liang, Liangjia Zeng, Ruoyun Zhou, Manting Feng, Yemin Li, Guangyao Hua, Huying Rao, Xiaozhen Lin, Min Yi

**Affiliations:** ^1^ Department of Endocrinology, The Second Affiliated Hospital of Guangzhou Medical University, Guangzhou, China; ^2^ Cardiovascular Medicine and Cardio-Oncology Group, Medical Exploration and Translation Team, Guangzhou, China; ^3^ Department of Clinical Medicine, The Sixth Affiliated Hospital of Guangzhou Medical University (Qingyuan People's Hospital), Qingyuan, China; ^4^ Department of Anesthesiology, The Second Clinical College of Guangzhou Medical University, Guangzhou, China; ^5^ Department of Medical Imageology, The Second Clinical College of Guangzhou Medical University, Guangzhou, China; ^6^ Department of Clinical Medicine, The Third Clinical College of Guangzhou Medical University, Guangzhou, China; ^7^ Department of Clinical Medicine, The Nanshan Clinical College of Guangzhou Medical University, Guangzhou, China; ^8^ Department of Clinical Medicine, The First Clinical College of Guangzhou Medical University, Guangzhou, China; ^9^ Department of Cardiology, The Sixth Affiliated Hospital of Guangzhou Medical University (Qingyuan People’s Hospital), Qingyuan, China; ^10^ Department of Cardiology, Guangzhou Institute of Cardiovascular Disease, The Second Affiliated Hospital of Guangzhou Medical University, Guangzhou, China

**Keywords:** cardiovascular disease death, cardio-oncology, prostate cancer, risk stratification, SEER

## Abstract

**Purpose:**

The risk of cardiovascular disease (CVD) mortality in patients with localized prostate cancer (PCa) by risk stratification remains unclear. The aim of this study was to determine the risk of CVD death in patients with localized PCa by risk stratification.

**Patients and methods:**

Population-based study of 340,806 cases in the Surveillance, Epidemiology, and End Results (SEER) database diagnosed with localized PCa between 2004 and 2016. The proportion of deaths identifies the primary cause of death, the competing risk model identifies the interaction between CVD and PCa, and the standardized mortality rate (SMR) quantifies the risk of CVD death in patients with PCa.

**Results:**

CVD-related death was the leading cause of death in patients with localized PCa, and cumulative CVD-related death also surpassed PCa almost as soon as PCa was diagnosed in the low- and intermediate-risk groups. However, in the high-risk group, CVD surpassed PCa approximately 90 months later. Patients with localized PCa have a higher risk of CVD-related death compared to the general population and the risk increases steadily with survival (SMR = 4.8, 95% CI 4.6–5.1 to SMR = 13.6, 95% CI 12.8–14.5).

**Conclusions:**

CVD-related death is a major competing risk in patients with localized PCa, and cumulative CVD mortality increases steadily with survival time and exceeds PCa in all three stratifications (low, intermediate, and high risk). Patients with localized PCa have a higher CVD-related death than the general population. Management of patients with localized PCa requires attention to both the primary cancer and CVD.

## Introduction

Prostate cancer (PCa) is the most common malignancy in the US and the second leading cause of death in men with an estimated 268,490 new cases and 34,500 deaths in 2022 (1). PCa is dominated by localized stages (approximately 70%), and risk stratification in localized stages can determine the risk of recurrence, reduce overdiagnosis, overtreatment and medical burden, and maximise benefits (morbidity and mortality) (1–5).

Improvements in PCa survival have focused attention on the competing causes of death, with a shift in the leading cause of death from cancer to non-cancer, particularly the predominance of cardiovascular disease (CVD) in non-cancer deaths (6–8). Patients with prostate cancer have a high burden of cardiovascular comorbidities (9), which is related to overlapping risk factors for cancer and CVD, cardiovascular toxicity of antineoplastic therapy, and cardiovascular risk factors are frequently underestimated and undertreated (10–15). Given that PCa is a highly heterogeneous disease, treatment decisions based on risk stratification will vary widely (16), resulting in differences in CVD risk exposure among patients with PCa.

Previous studies have mainly focused on the effects of treatment modalities on CVD-related risk in PCa patients (8, 17–19), and a few studies have focused on risk stratification (20). Similarly, the American Society of Clinical Oncology (ASCO) guidelines for the survivorship care of PCa support the assessment and screening of CVD risk factors in patients receiving anticancer treatment (21), but lack attention to CVD risk in PCa patients with different risk stratification. Based on the clinical, morphological, and molecular heterogeneity of PCa (22), some studies have focused on the cause of death in metastatic, locally advanced, and high-risk elderly PCa patients, but the results may not be applicable to PCa patients with different risk stratification (23, 24). Previous studies have suggested that the risk of CVD in PCa patients is related to risk stratification, but the conclusions are still controversial. Some studies have found that the risk of death from CVD exceeds the risk of death from PCa (25, 26), while others have found the opposite conclusion (20, 27). Therefore, further investigation and clarification of the risk of CVD-related mortality in risk stratified PCa patients is needed.

This study describes the competing risks of CVD-related death in localized PCa patients by risk stratification, and further quantifies the long-term and short-term CVD mortality of PCa patients compared to the general population across risk stratification. This study could provide population-level data to help guide the management and follow-up of risk stratified PCa patients.

## Materials and methods

### Data resources and patient selection

This study used data from the Surveillance, Epidemiology, and End Results (SEER) database were used in this study. The SEER program is the authoritative source for cancer registries conducted by national cancer registries, with high quality demographic and cancer-specific information and avoids surveillance bias through systematic, standardized and regular data collection procedures, covering approximately 30% of the population (28). All patients diagnosed with first primary PCa between 2004 and 2016 were considered in this study. The inclusion criteria were (1) histologic diagnosis between 2004 and 2016; (2) case selection (site and morphology, primary site-labeled) = “C61.9”; (3) single primary cancer; (4) definite cause of death and active follow-up; Exclusion criteria were (1) prostate-specific antigen (PSA) unknown; (2) missing Gleason score (GS); (3) missing TNM staging; (4) unknown race; (5) other than localized stage; (6) follow-up less than 2 months. PCa patients were divided into three risk stratification groups based on initial prostate-specific antigen (PSA) concentration, Gleason score (GS), and T-stage as described by D’Amico (29). The risk categories were defined as follows: (1) low risk was defined as PSA ≤ 10 and GS ≤ 6 and cT1c-2a; (2) intermediate risk was defined as PSA >10–20 or GS 7 or cT2b; (3) high risk was defined as PSA >20 or GS 8–10 or cT2c (29). Ethics committee approval was not required for publicly available data.

### Participant variables and outcomes

Patient variables included age at diagnosis (<65 years, 65–85 years, >85 years) (30), race (white, black, others), year of diagnosis (2004–2009, 2010–2016), survival month (2–11, 12–35, 36–59, 60–119, 120–179), grade (low, high, others, unknown), surgery (yes, no, unknown), radiotherapy (yes, no evidence) and chemotherapy (yes, no evidence).

The primary outcome was death from all causes, including PCa, other cancers, CVD, and other non-neoplastic, with cause of death based on physician certification. We classified causes of death in the SEER database according to the International Classification of Diseases 10 (ICD-10) of the National Cancer Health Statistics (28). Person-years of follow-up were cumulated started from diagnosis of PCa and ended at the date of death, loss to follow-up, or the date of final follow-up (December 31, 2018).

### Statistical analyses

The distribution of baseline characteristics in the three risk stratification groups was described by component ratios. Chi-square tests were used to evaluate the comparison of two or more component ratios. The distribution of causes of death will be presented as percentages, with the proportion of deaths defined as the number of specific causes of death divided by the total number of deaths in PCa patients. To further assess the interaction between PCa, CVD-related deaths and other causes of death among PCa patients, a competing risk models were used to estimate the crude cumulative mortality and further plotted according to risk stratification (31, 32). Standardized mortality ratios (SMR) were used to compare the CVD-related mortality among PCa patients with the general male population, stratified according to risk stratification and characteristics (33). The SMR was calculated as the ratio of observed specific deaths to the number of expected deaths, while the expected number of deaths was calculated according to the formula: expected deaths = person-years × CVD mortality rate in the general population. The mortality rate of CVDs among general population is available on CDC WONDER, while the person-years is the sum of survival times from diagnosis of PCa to date of CVD or study completion. The SMRs were calculated with a 95% confidence interval for CVD-related mortality using the methods mentioned before (34). All statistical analyses were completed with R software (version 3.4.4). Significance was defined by a *P*-value <0.05.

## Results

### Participant characteristics

A total of 340,806 patients diagnosed with PCa were included in this study. The median follow-up time was 6.5 years [interquartile range (IQR) 3.6–9.4]. 51.9% were aged 65–84 (Table 1), 77.6% were white, 52.4% were diagnosed between 2010 and 2016, 53.3% had low-grade tumors, 62.1% did not undergo surgery, 60.0% did not receive radiotherapy and 99.8% did not receive chemotherapy. PCa patients in the low- and intermediate-risk groups were predominantly aged 65–84 (50.6% and 60.4%, respectively), while those in the high-risk group were predominantly aged <65 (49.8%). Similar proportions of low- and intermediate-risk patients underwent surgery (15.2% and 15.4%, respectively), while high-risk patients were significantly higher (60.0%). Patients with intermediate-risk PCa are more likely to receive radiotherapy (61.8%), and the vast majority of all risk stratifications do not receive chemotherapy (99.8%). PCa patients in the low-risk group were dominated by low-grade tumors (97.1%), while those in the intermediate- and high- risk groups were dominated by high-grade tumors (56.4% and 62.4%, respectively).

**Table 1:** Baseline characteristics of patients with prostate cancer by risk stratifications.

Characteristic	Risk stratifications (*n*/%)				*P* value
	Total	Low risk	Intermediate risk	High risk	
Overall	340,806	93,617	84,256	162,933	
Age at diagnosis, years					<0.001
<65	1,57,291 (46.2)	45,696 (48.8)	30,417 (36.1)	81,178 (49.8)	
65–85	1,76,753 (51.9)	47,412 (50.6)	52,593 (62.4)	76,748 (47.1)	
>85	6,762 (2.0)	509 (0.5)	1,246 (1.5)	5,007 (3.1)	
Race					<0.001
White	2,64,585 (77.6)	74,413 (79.5)	63,155 (75.0)	1,27,017 (78.0)	
Black	57,968 (17.0)	14,592 (15.6)	16,429 (19.5)	26,947 (16.5)	
Others^a^	18,253 (5.4)	4,612 (4.9)	4,672 (5.5)	8,969 (5.5)	
Year of diagnosis					<0.001
2004–2009	1,62,087 (47.6)	45,720 (48.8)	36,170 (42.9)	80,197 (49.2)	
2010–2016	1,78,719 (52.4)	47,897 (51.2)	48,086 (57.1)	82,736 (50.8)	
Survival months					<0.001
2–11	24,321 (7.1)	6,395 (6.8)	7,909 (9.4)	10,017 (6.1)	
12–35	58,441 (17.1)	13,657 (14.6)	15,741 (18.7)	29,043 (17.8)	
36–59	59,754 (17.5)	15,678 (16.7)	15,512 (18.4)	28,564 (17.5)	
60–119	1,45,186 (42.6)	41,041 (43.8)	34,127 (40.5)	70,018 (43.0)	
120–179	53,104 (15.6)	16,846 (18.0)	10,967 (13.0)	25,291 (15.5)	
Grade					<0.001
Low	1,81,779 (53.3)	90,873 (97.1)	35,991 (42.7)	54,915 (33.7)	
High	1,51,026 (44.3)	1,841 (2.0)	47,550 (56.4)	1,01,635 (62.4)	
Others^b^	29 (0.0)	0 (0.0)	0 (0.0)	29 (0.0)	
Unknown	7,972 (2.3)	903 (1.0)	715 (0.8)	6,354 (3.9)	
Surgery					<0.001
Yes	1,25,053 (36.7)	14,263 (15.2)	12,959 (15.4)	97,831 (60.0)	
No	2,11,516 (62.1)	77,702 (83.0)	70,018 (83.1)	63,796 (39.2)	
Unknown	4,237 (1.2)	1,652 (1.8)	1,279 (1.5)	1,306 (0.8)	
Radiotherapy					<0.001
Yes	1,36,178 (40.0)	42,808 (45.7)	52,086 (61.8)	41,284 (25.3)	
No	2,04,628 (60.0)	50,809 (54.3)	32,170 (38.2)	1,21,649 (74.7)	
Chemotherapy					<0.001
Yes	830 (0.2)	80 (0.1)	132 (0.2)	618 (0.4)	
No	3,39,976 (99.8)	93,537 (99.9)	84,124 (99.8)	1,62,315 (99.6)	

^a^ Other includes American Indian/Alaska Native and Asian/Pacific Islander.

^b^ Other includes B-cell, pre-B, B-precursor and B-cell.

### Proportion of deaths

**Figure 1:**
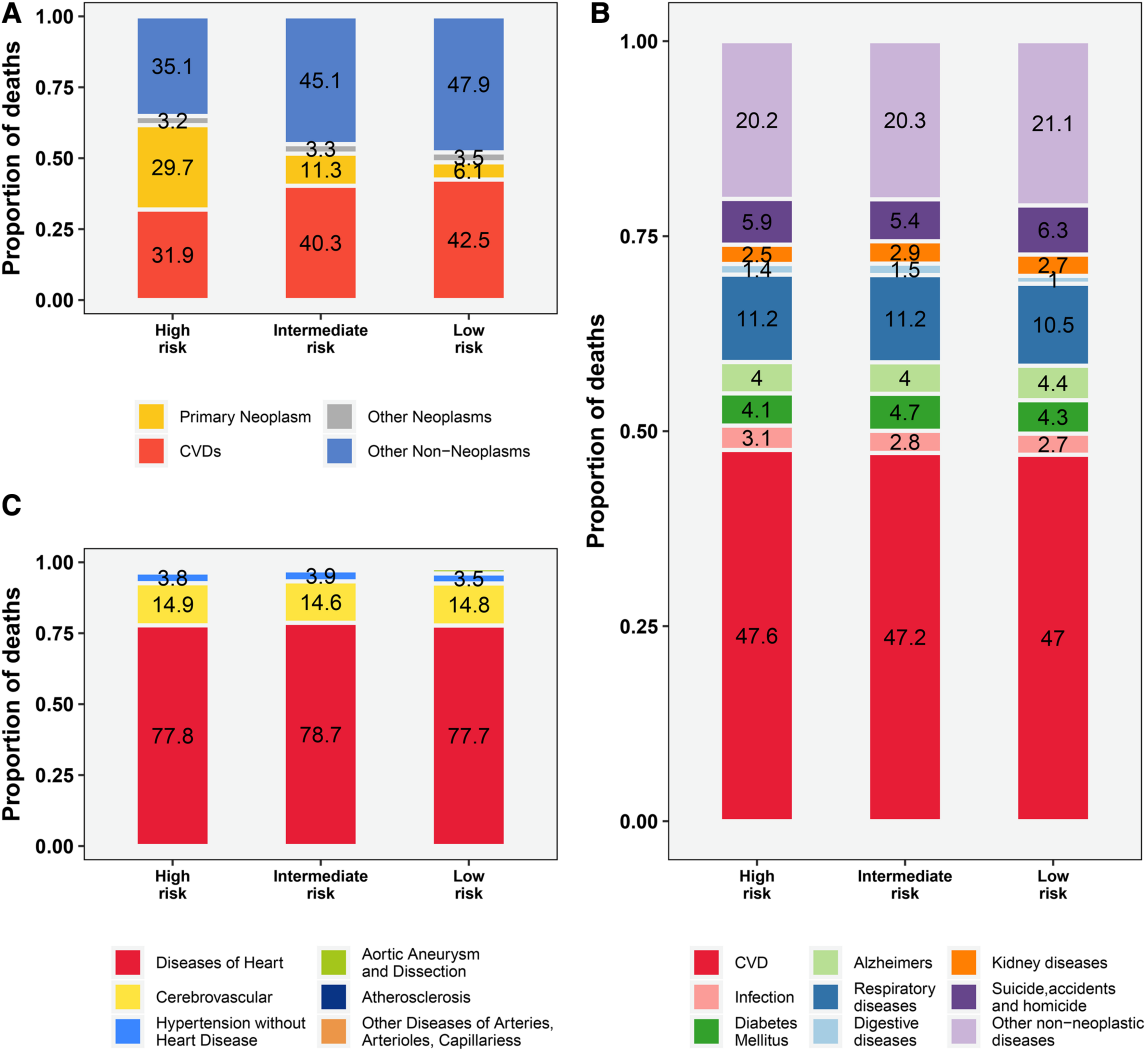
The proportion of deaths among patients with prostate cancer by risk stratification. (**A**) all causes of deaths; (**B**) causes of non-cancer deaths; (**C**) causes of CVD-related deaths. The proportion of other CVD deaths (including Aortic Aneurysm and Dissection, Atherosclerosis, Other Diseases of Arteries, Arterioles, Capillaries) is not shown in the figure with specific numbers (vary from 0.7% to 1.9%, Figure 1C). CVD, cardiovascular disease.

The proportion of deaths from primary malignancy (PCa) gradually decreases from high-risk to low-risk subpopulations (29.7%–6.1%, Figure 1A), while the proportion of deaths from CVD and other non-cancer causes exceeds that of PCa in all 3 risk stratifications, especially in the low-risk subgroup (90.4% vs. 6.1%, Figure 1A). Among all non-cancer causes of death, CVD accounted for the highest proportion (47%–47.6%, Figure 1B). Among all CVD deaths, heart disease dominated (77.7%–78.7%, Figure 1C), followed by cerebrovascular disease (14.6%–14.9%, Figure 1C) and hypertension without heart disease (3.5%–3.9%, Figure 1C).

### Cumulative mortality

**Figure 2:**
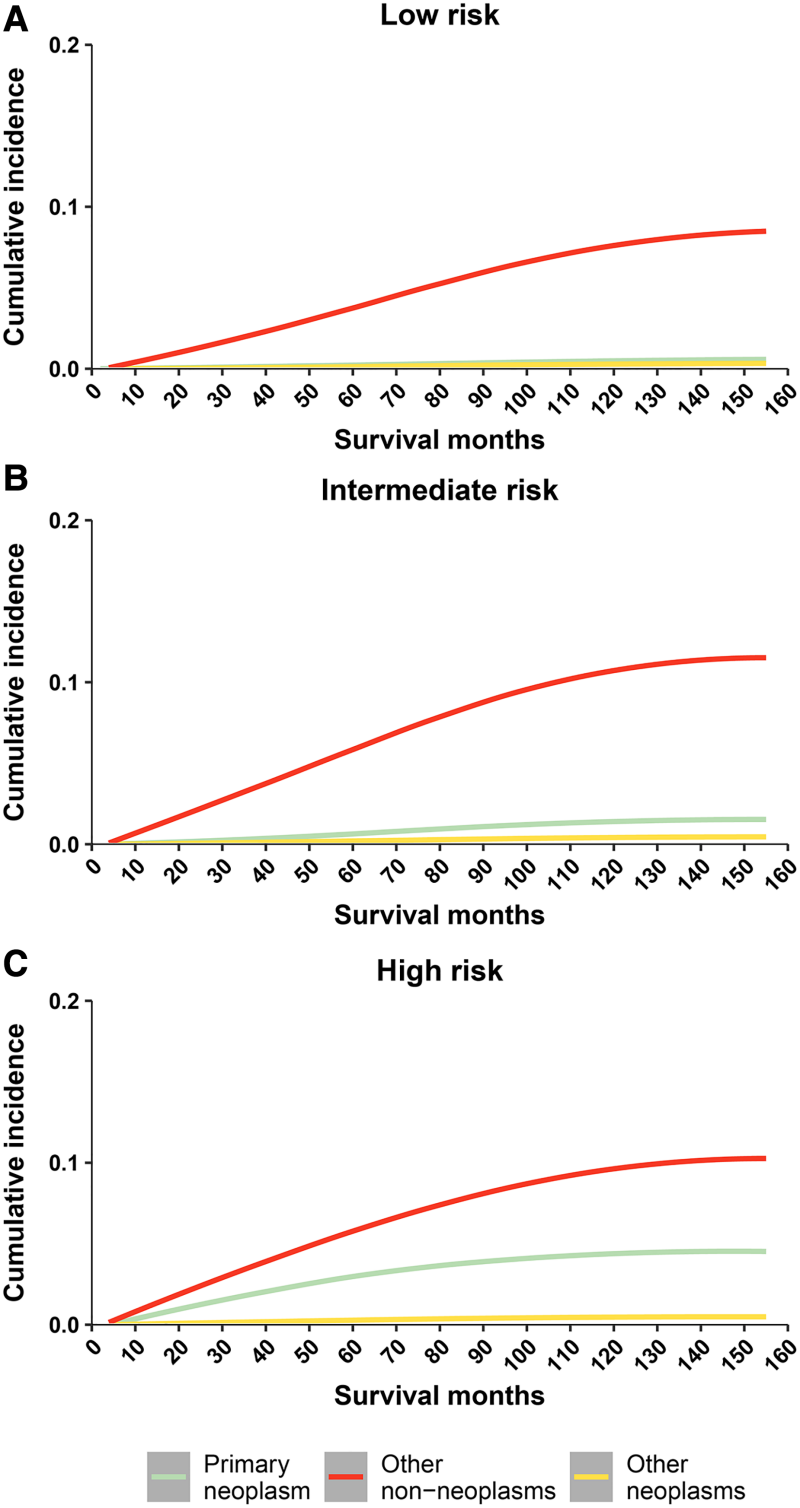
Cumulative mortality among patients with prostate cancer by risk stratification (three causes of death include: primary neoplasm, other non neoplams, and other neoplams). (**A**) cumulative risk of death in low-risk prostate cancer patients; (**B**) cumulative risk of death in intermediate-risk prostate cancer patients; (**C**) cumulative risk of death in high-risk prostate cancer patients.

**Figure 3:**
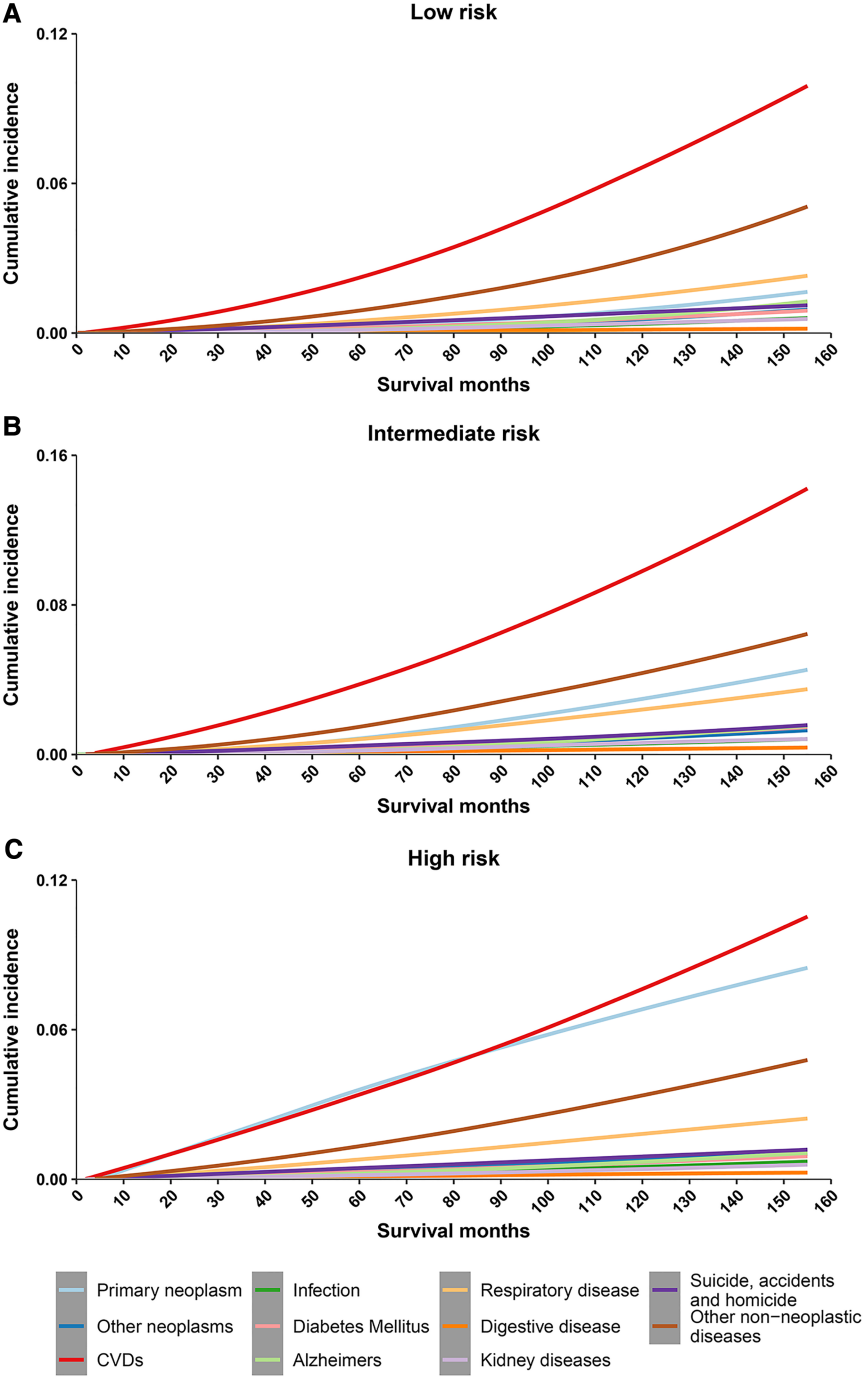
Cumulative mortality among patients with prostate cancer by risk stratification (with detailed classification of nonneoplasms deaths). (**A**) cumulative risk of death in low-risk prostate cancer patients; (**B**) cumulative risk of death in intermediate-risk prostate cancer patients; (**C**) cumulative risk of death in high-risk prostate cancer patients. CVD, cardiovascular disease.

Cumulative mortality for other non-neoplasms increased steadily with survival time, and overtaking for cancer was observed in all 3 risk stratifications (Figure 2). Cumulative mortality from other non-cancer causes was further subdivided. The risk of CVD consistently exceeded that of the primary neoplasm in the low- and intermediate- risk groups almost at the same time as PCa was diagnosed (Figures 3A,B). In the high-risk group, CVD overtook primary neoplasms approximately 90 months after cancer diagnosis (Figure 3C). Subcategories of CVD have also been used in competing risk studies, and the risk of heart disease surpasses primary neoplasm in both the low- and intermediate risk groups (Supplementary Figures S1A,S1B). The cumulative risk of primary neoplasm consistently leads in the high-risk group, followed by heart disease (Supplementary Figure S1C). Cumulative risks for various clinical characteristics were also calculated, and the risk of CVD exceeded that of PCa in the vast majority of subgroups (Supplementary Figures S2–S7), except for high-risk groups aged <65 years (Supplementary Figure S2G) and high-risk groups diagnosed between 2010 and 2016 (Supplementary Figure S4G). The cumulative risk of CVD, which was consistently higher than that of PCa, was observed in the high-risk radiotherapy group, whereas the excess of CVD in the non-radiotherapy group occurred approximately 100 months after diagnosis (Supplementary Figures S7E,S7F).

### Mortality compared with the general population

**Figure 4:**
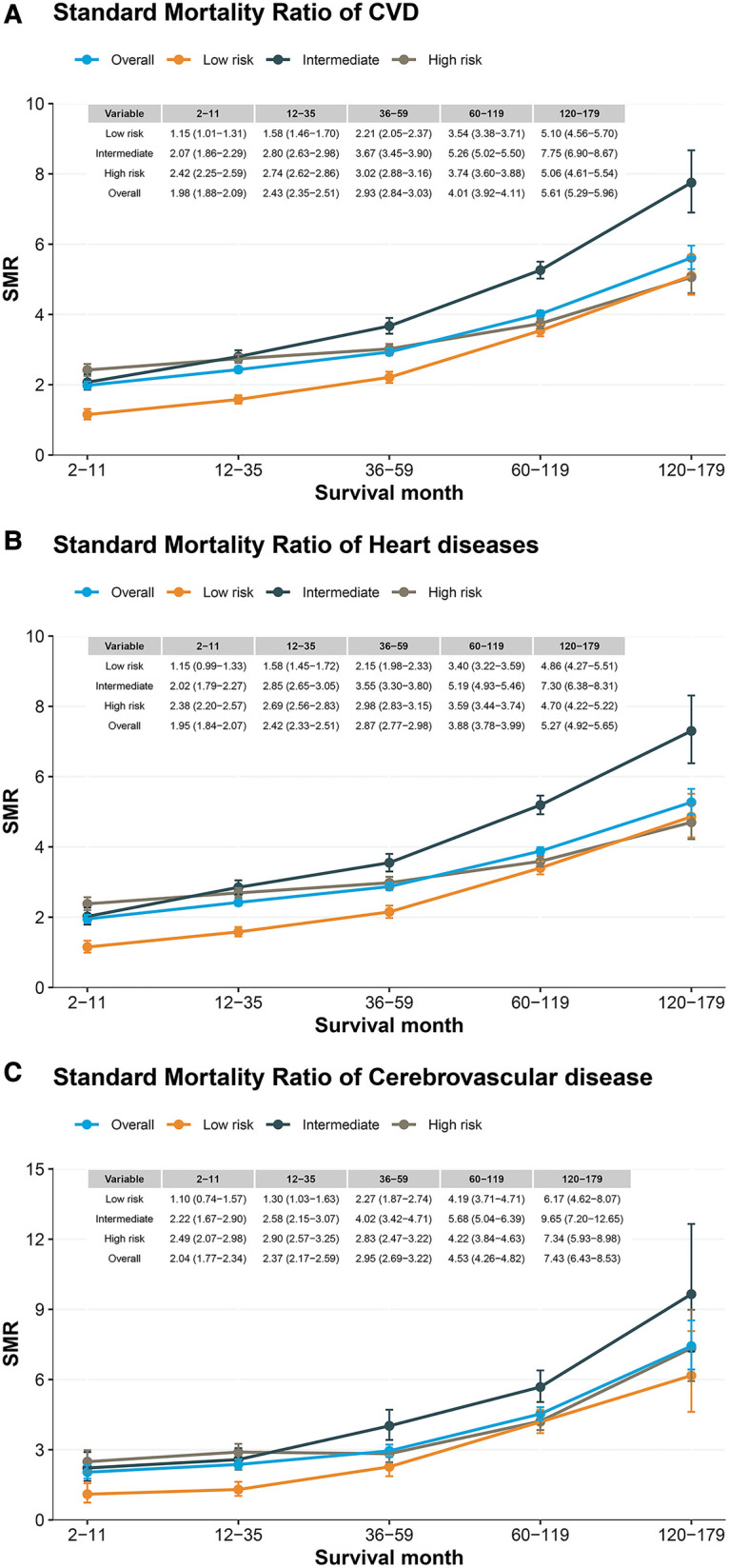
Standard mortality ratio among patients with prostate cancer by risk stratification. (**A**) CVD standard mortality ratio in patients with localized prostate cancer; (**B**) heart disease standard mortality ratio in patients with localized prostate cancer; (**C**) Cerebrovascular disease standard mortality ratio in patients with localized prostate cancer.

Compared to the general population, patients with PCa had a higher risk of CVD death, which increased with longer follow-up (Figure 4A). In contrast to the low-risk group, the intermediate- and high- risk groups have a higher risk of CVD death, and the intermediate risk group increases more significantly with the follow-up time (SMR: from 2.07 to 7.75, Figure 4A). Similar results were observed for heart disease and cardiovascular disease (Figures 4B,C). In subgroup analyses, we observed similar results in subpopulations, including age at diagnosis, race, year of diagnosis, surgery, radiation, and grade (Supplementary Table S1).

## Discussion

This large-scale, population-based and long-follow-up study provides a comprehensive assessment of the risk of CVD-related death in localized PCa patients under risk stratification. To our knowledge, this is the first study in localized PCa to focus on CVD-related deaths under risk stratification. Our results show that the risk of CVD-related death increases steadily with prolonged survival in all three PCa risk stratifications and surpasses PCa as the leading cause of death. Consistent conclusions were reached in subpopulation analyses, and we also observed a higher risk of CVD-related death in patients with localized PCa compared to the general population.

Our findings were confirmed in both death-proportion and competing risk models, echoing the controversy of previous studies regarding the risk of CVD mortality in PCa patients. A retrospective study (20) that included patients with non-metastatic PCa did not observe CVD-related deaths over PCa, which may be attributed to the pooling of patients with PCa masking heterogeneous survival outcomes at different stages. On the contrary, some studies (23, 25, 26) support our finding that the risk of CVD-related death exceeds that of PCa, however, they either focus mainly on PCa patients under specific treatments, or only focus on a certain age group, lacking analysis under different risk stratification. Given that the current ESMO consensus recommends adequate cardiovascular assessment before anticancer therapy (35), studies on the risk of CVD-related death in PCa patients undergoing risk stratification are warranted.

We found that the risk of CVD-related death surpassing PCa varied by risk stratification. In the low- and intermediate-risk groups, the risk of CVD-related death exceeded that of PCa almost at the time of diagnosis, but was delayed until about 90 months in the high-risk group. Several factors may help explain this. First, active surveillance is recommended for PCa patients in the low- and some intermediate-risk groups (16). A clinical study (36) observed that CVD deaths in active surveillance PCa patients were approximately 3 times higher than primary cancer, which may be explained by the low malignancy of the cancer leading to long survival times, implying continued exposure to common risk factors for CVD and cancer (6, 11). Second, in the high-risk group had more aggressive cancer progression, poorer prognosis, and higher risk of cancer-specific death (27). Third, treatment modalities are more complex in the high-risk group (16), and anticancer therapy may increase CVD risk (25, 26, 37), perhaps explaining the progressively higher risk of CVD-related death and surpassing PCa in the high-risk group.

Risk stratification of PCa can help predict the probability of biochemical recurrence after local therapy, however, as suggested by the NCCN guidelines, there is still heterogeneity and prognostic differences within risk groups (38). Considering that data analysis of only risk-stratified PCa patients may obscure information about subgroup characteristics, we performed a subgroup analysis of risk-stratified PCa patients. We found that all subgroups in the low- and intermediate-risk groups (including age, race, surgery, year of diagnosis, pathological grade, radiotherapy) had a higher risk of CVD-related death than PCa. This finding is partly supported by a study focusing on non-curative treatments for PCa patients, which observed similar trends in the low- and intermediate-risk groups age ≥65, but not in those age <65 (27). This may be due to differences in the study populations, but also means that their findings are difficult to generalize to the populations of interest to us. Notably, not all subgroups of CVD exceeded PCa, and PCa was consistently higher than CVD in high-risk PCa patients aged <65 years, suggesting that management of PCa remains a major concern despite the non-negligible risk of CVD-related death. Our study applied PCa risk stratification to cardiovascular risk assessment, which may provide a basis for individualized risk stratification and a more refined, individualized assessment reference.

We further quantified the risk of CVD-related death in PCa patients in different risk stratifications. Compared with the general population, PCa patients in all three risk stratifications had a higher risk of CVD-related death, which is consistent with a study focusing on short-term follow-up of PCa patients, with the substantially higher risk of CVD in the first month (39). Further support comes from another study using SMR, which found that patients with cancer of all sites, including PCa, consistently had a higher risk of death from CVD than the general population (40). However, another study observed a lower risk of CVD death than the general population (7), which may be explained by combining both localized and regional PCa populations, ignoring the prognostic differences between them (6). We also found that the risk of CVD-related death was higher in the intermediate- and high- risk groups compared with the low-risk group, and was most pronounced in the intermediate-risk group with increasing follow-up time. This is supported by a retrospective analysis focusing on PCa patients treated with ADT (41) that observed an excess risk of cardiac death in intermediate-risk PCa patients compared with the low-risk group. There could be several possible explanations, first, as they expressed, they found that the intermediate risk group was older and therefore had a higher risk of CVD death, which was also observed in our study. Second, compared with low-risk PCa patients diagnosed by PSA screening, the intermediate-risk population is less health-conscious (42) and may have more CVD risk factors and comorbidities. Third, compared with low-risk PCa patients, intermediate-risk patients receive more aggressive treatments (16), which may be associated with cardiotoxicity.

The traditional treatment approach prioritizes cancer treatment, but attention must also be paid to the competing risks associated with cancer, especially CVD, which may provide a further step in survival. An RCT study of ADT couldn’t even get enough expected events because of the involvement of cardiologists to control for risk factors, making shared management especially important (43). This should be of interest to multidisciplinary teams working together to manage and reduce the risk of CVD death in PCa patients. As recommended by the ESC guidelines on cardio-oncology (44), practical cardioprotective strategies should be developed and implemented, including optimization of common risk factors, active surveillance, adjustment of dose and infusion time of anticancer drugs, and maintenance of adequate physical activity (45).

Although the mechanisms underlying the elevated risk of CVD in patients with PCa remain controversial, these factors may help explain. These include cardiotoxicity of anticancer therapy, aging, immediate psychological stress, common risk factors for CVD and cancer, and cardiovascular damage from PCa. First, several studies have suggested that anticancer treatment may be associated with coronary artery disease, myocardial infarction, sudden cardiac death and metabolic syndrome, which can increase the risk of CVD-related death (10, 46). Second, vascular changes occur subsequently with age, including central artery stiffness and systemic endothelial dysfunction (47), and most PCa occurs in the elderly (23), with a correspondingly increased risk of CVD-related death. Third, PCa shares common risk factors with CVD, including smoking and diabetes, which are recognized to increase the risk of CVD-related death (11). Fourth, the diagnosis of PCa can cause immediate psychological stress and trigger sudden cardiac arrest, which can manifest as various forms of arrhythmias, myocardial infarction, and sudden death (39, 48). Fifth, PCa may induce arterial-venous thromboembolism and increase the risk of cardiovascular events such as stroke (49).

## Limitations

Several limitations of the study should be considered. First, the SEER database does not provide specific treatment information, comorbidities, risk factors, and demographic characteristics, which limits our further analysis, evaluation, and generalization of conclusions (50). Second, cause of death may be misclassified, but it is negligible. Third, given the wide period of this retrospective study, some confounding factors are inevitable. For example, the treatment and management of PCa and CVD have changed during follow-up and can, therefore, confound the results.

## Conclusions

CVD-related death is the primary competing risk in patients with localized PCa. In the low- and intermediate-risk groups, the risk of CVD death exceeded that of PCa almost as soon as PCa was diagnosed, whereas in the high-risk group, the excess of CVD death risk occurred at approximately 90 months. In all 3 risk stratifications, PCa patients have a higher risk of CVD-related death than the general population. These results highlight differences in CVD mortality risk between PCa risk stratifications and may provide insights into cardiac oncology care, detection, screening, prevention, and treatment strategies for PCa patients.

## Data Availability

Publicly available datasets were analyzed in this study. This data can be found here: http://seer.cancer.gov.
