# Supplementary material for: Cardiovascular mortality by cancer risk stratification in patients with localized prostate cancer: a SEER-based study
Source: Front Cardiovasc Med. 2023 Aug 4;10:1130691. doi: 10.3389/fcvm.2023.1130691 (PMC10443648; doi:10.3389/fcvm.2023.1130691)
Supplement: Supplementary file 2 [file Table2.docx]

Supplementary Material

**Cardiovascular Mortality among Patients with Localized Prostate Cancer** **by** **Risk Stratification**

**Zehao Luo**

*** Correspondence:** Xiaozhen Lin, MD, PhD: [linxz021@163.com](mailto:%20linxz021@163.com); Min Yi, MD, PhD: [smu_min@126.com](mailto:smu_min@126.com).

**List of Supplementary Table**

**Table S1**. Standard mortality ratio among patients with prostate cancer by risk stratification in subpopulations (in Excel Table).

Abbreviations: CVD, cardiovascular disease.

**List of Supplementary Figures**

**eFigure S1**. Cumulative mortality among patients with prostate cancer by further classification of cardiovascular death and non−neoplasms deaths.

**eFigure S2**. Cumulative mortality among patients with prostate cancer by age groups.

**eFigure S3**. Cumulative mortality among patients with prostate cancer by race groups.

**eFigure S4**. Cumulative mortality among patients with prostate cancer grouped by year of diagnosis.

**eFigure S5**. Cumulative mortality among patients with prostate cancer by grade.

**eFigure S6**. Cumulative mortality among patients with prostate cancer by surgery groups.

**eFigure S7**. Cumulative mortality among patients with prostate cancer by radiotherapy groups.


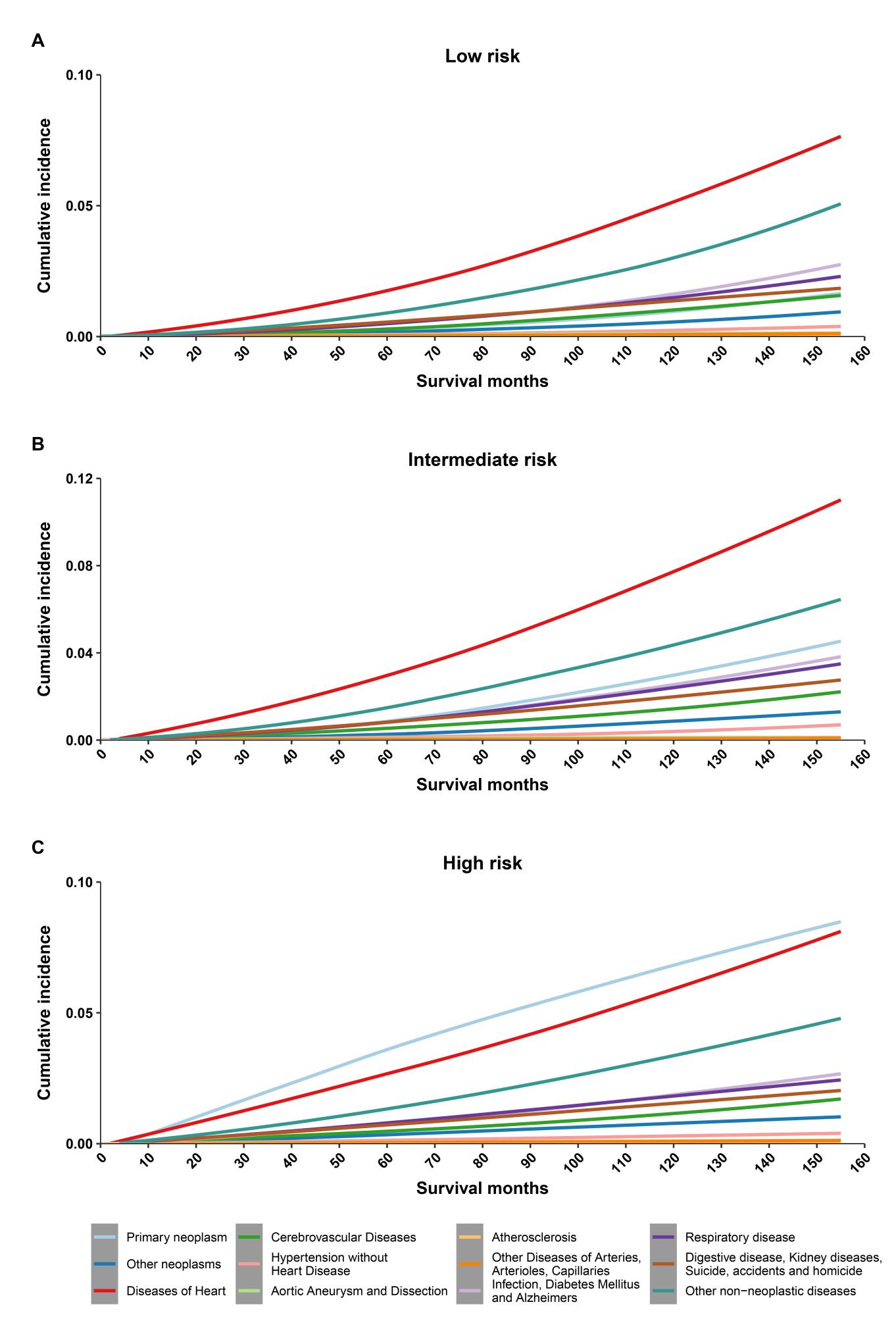


**eFigure S1**. Cumulative mortality among patients with prostate cancer by further classification of cardiovascular death and non−neoplasms deaths.


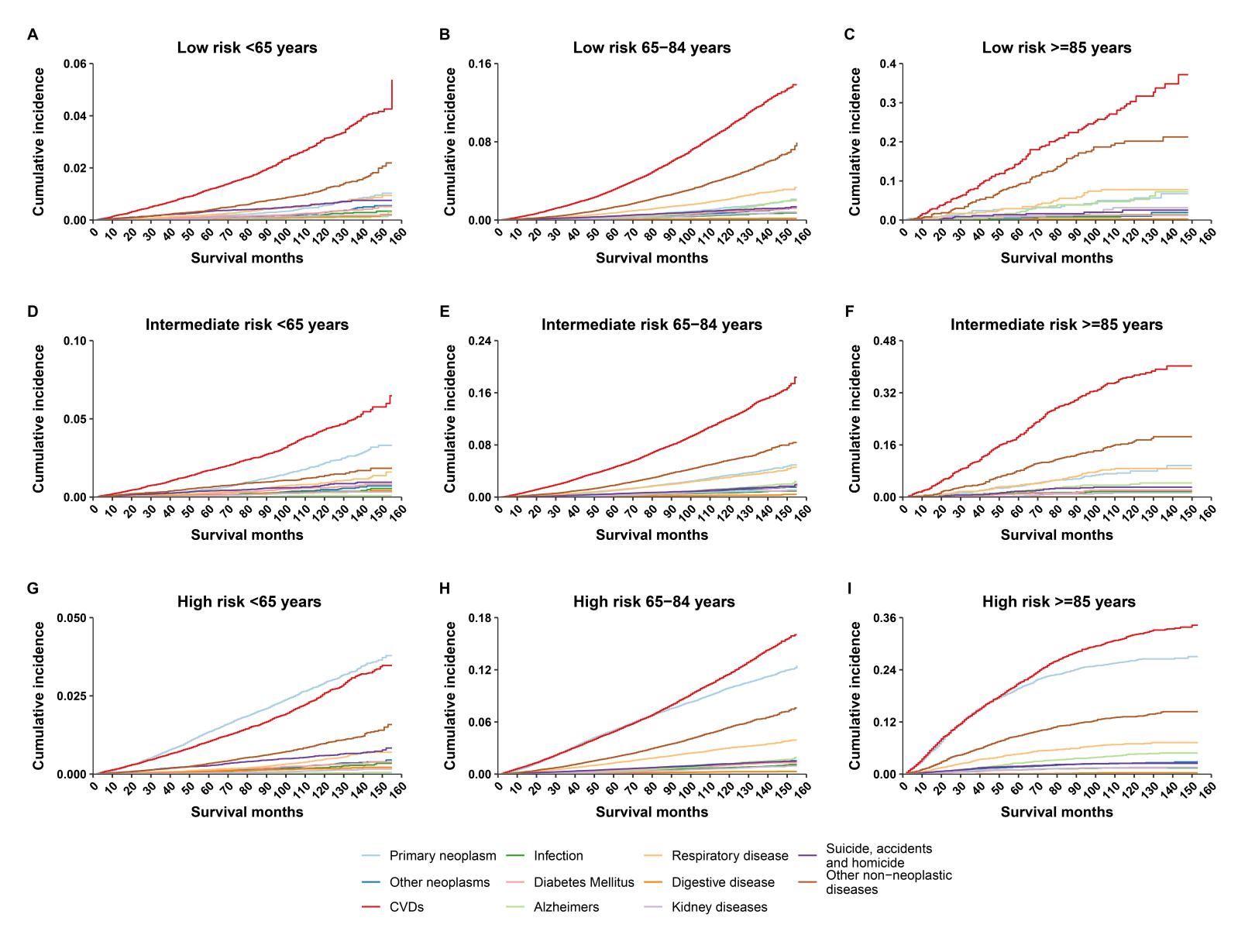
**eFigure S2**. Cumulative mortality among patients with prostate cancer by age groups.


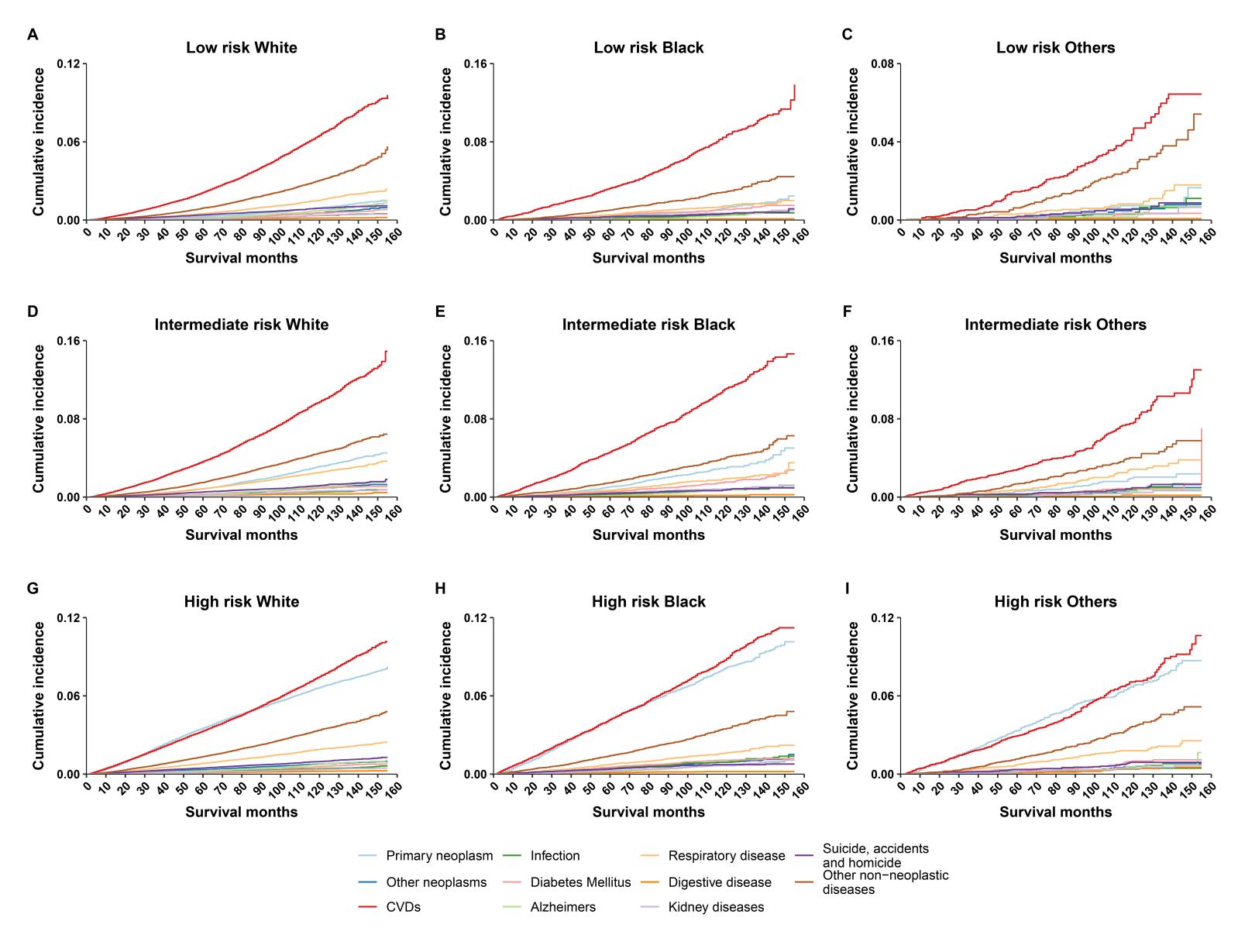


**eFigure S3**. Cumulative mortality among patients with prostate cancer by race groups.


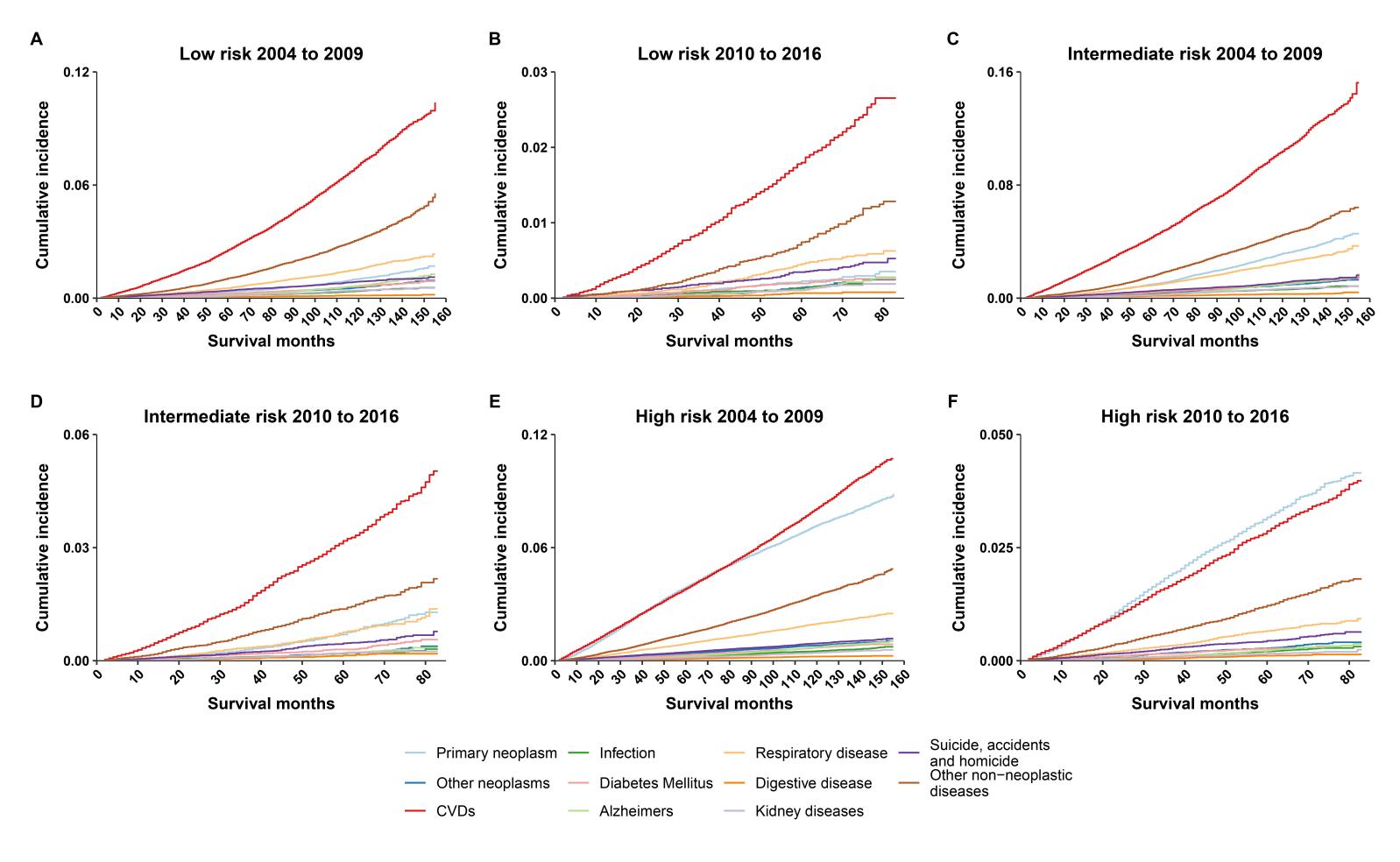


**eFigure S4**. Cumulative mortality among patients with prostate cancer grouped by year of diagnosis.


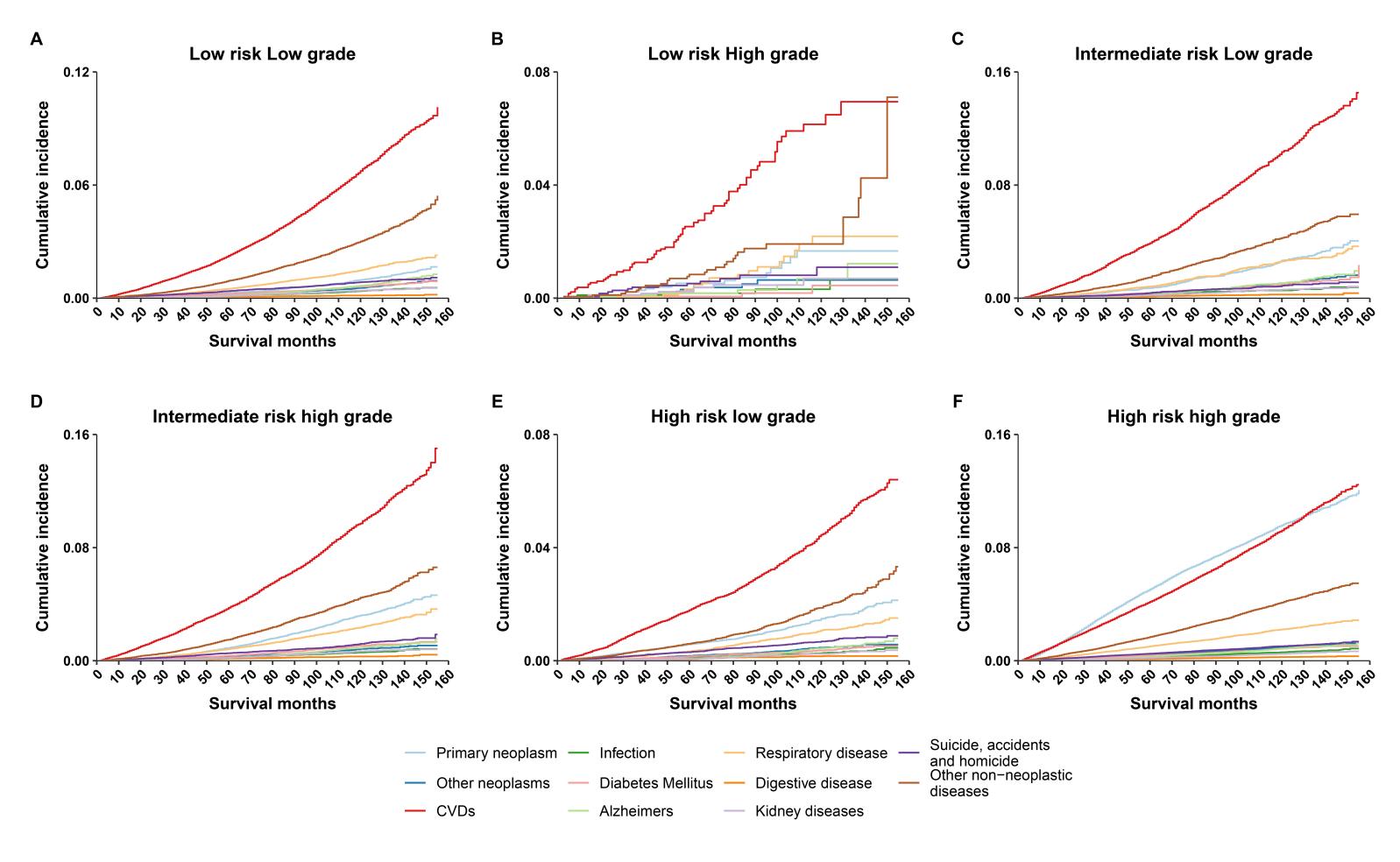


**eFigure S5**. Cumulative mortality among patients with prostate cancer by grade.


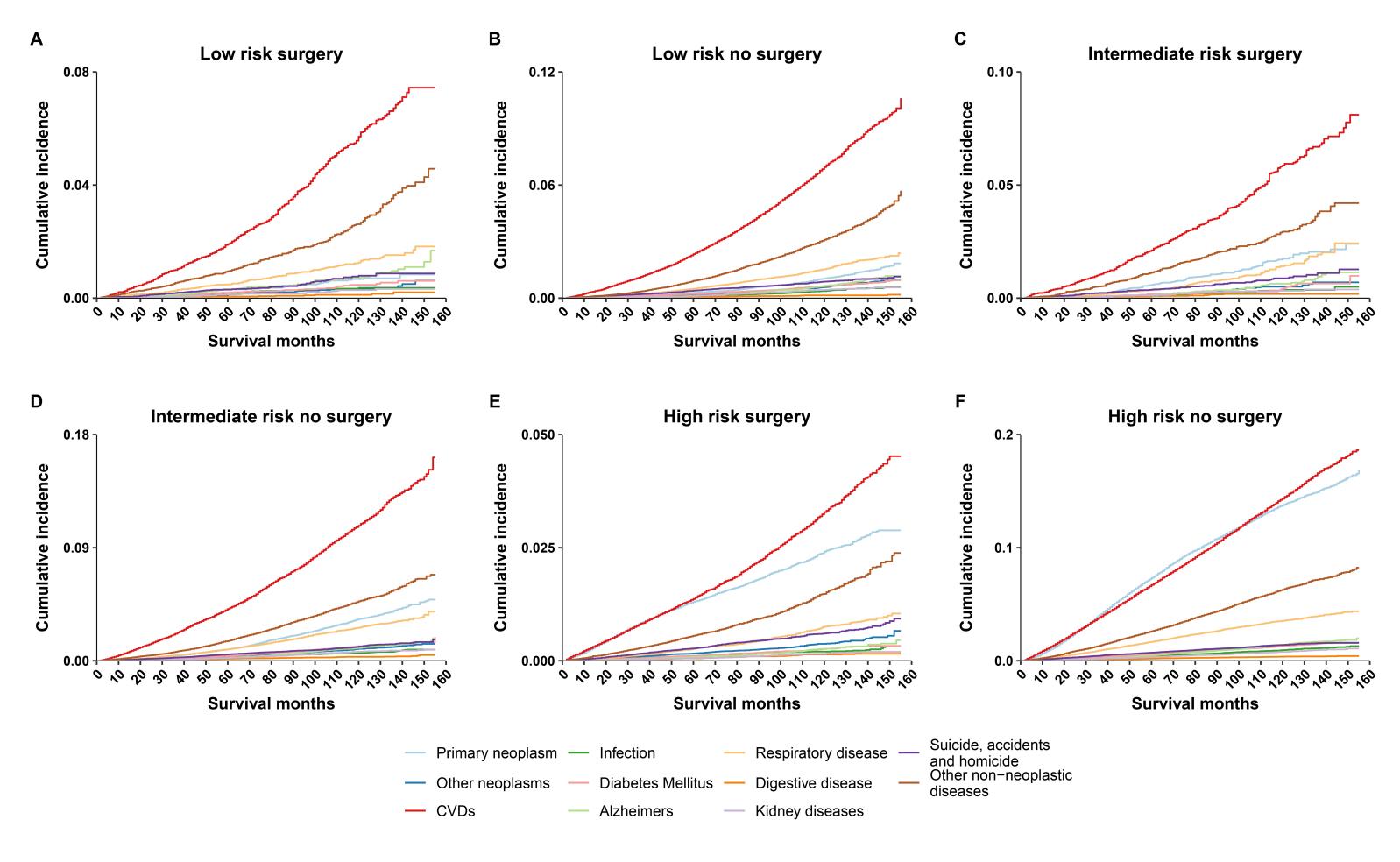


**eFigure S6**. Cumulative mortality among patients with prostate cancer by surgery groups.


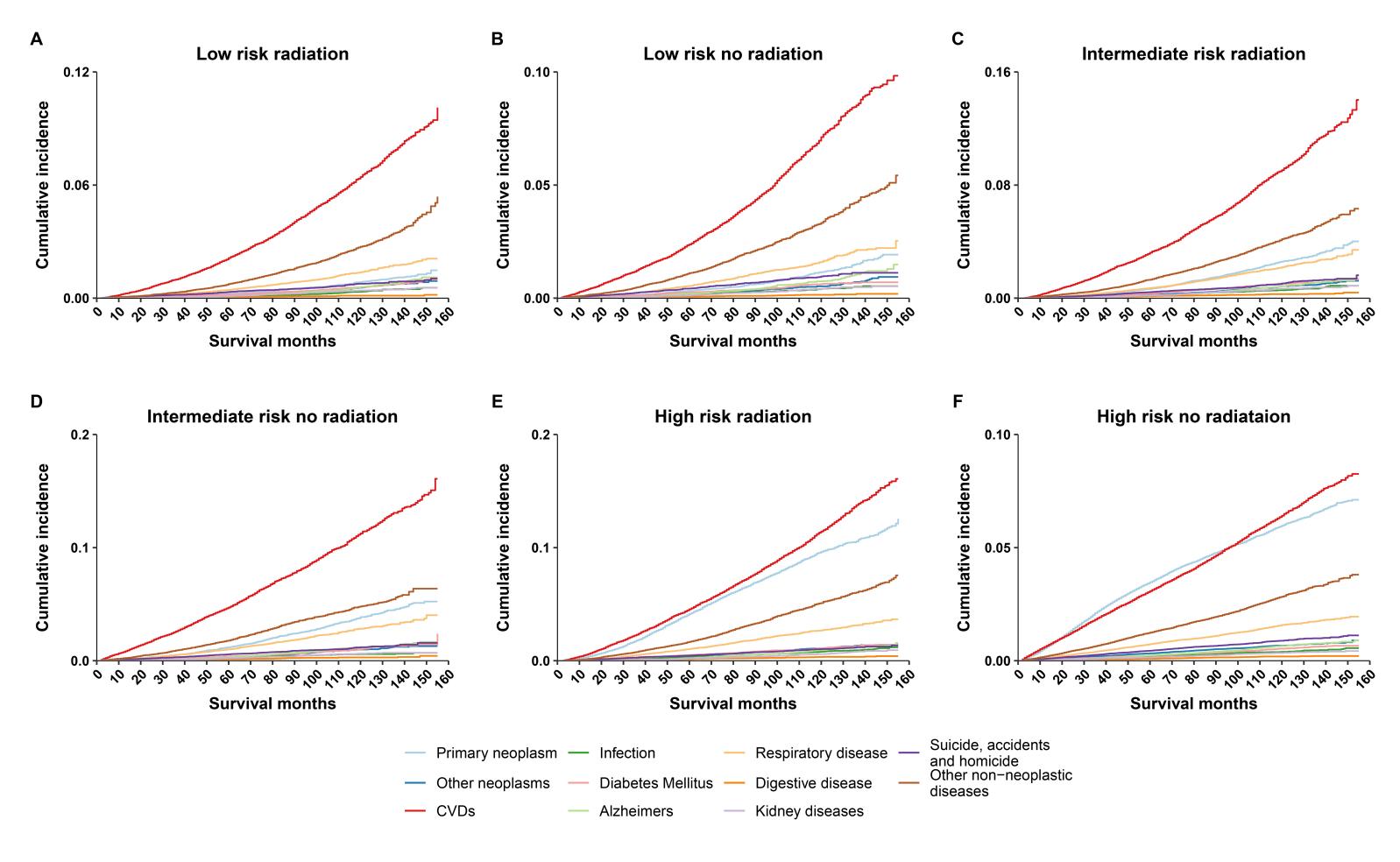


**eFigure S7**. Cumulative mortality among patients with prostate cancer by radiotherapy groups.
